# Pleural Marginal Zone Lymphoma Masquerading as Metastatic Adenocarcinoma of the Lung

**DOI:** 10.7759/cureus.73462

**Published:** 2024-11-11

**Authors:** Nicole Onatsko, Imad Karam, Aye Thida, Hagar Attia, Dedipya Bhamidipati, Rachelle Hamadi, Raavi Gupta, Mohan Preet

**Affiliations:** 1 Hematology and Oncology, State University of New York (SUNY) Downstate Health Sciences University, Brooklyn, USA; 2 Pathology and Laboratory Medicine, State University of New York (SUNY) Downstate Health Sciences University, Brooklyn, USA; 3 Internal Medicine, State University of New York (SUNY) Downstate Health Sciences University, Brooklyn, USA

**Keywords:** chronic inflammation, extranodal marginal zone lymphoma, pleural aspergillus, pleural effusion, pleural marginal zone lymphoma, primary lung adenocarcinoma

## Abstract

Extranodal marginal zone lymphoma (EMZL) is a rare subtype of non-Hodgkin's lymphoma characterized by the malignant transformation of lymphoid tissue at sites affected by chronic inflammation. Pleural marginal zone lymphoma (PMZL) is an infrequent manifestation of this condition. We report a case of PMZL co-occurring with primary lung adenocarcinoma.

This case involves an 88-year-old female patient who presented to the emergency department with recurrent pleural effusions and symptoms suggestive of decompensated heart failure. A thoracentesis of the effusion revealed an aspergillus population. Throughout her hospitalization, the patient underwent multiple evaluations for malignancy; however, no conclusive findings emerged. Ultimately, PMZL and poorly differentiated primary adenocarcinoma of the lung were confirmed through random biopsies of the parietal pleura and lung opacities, respectively. The pleural pathology showed a monoclonal population of immunoglobulin G kappa, positive for cluster of differentiation (CD) markers CD20 and CD43. Consequently, she was treated with rituximab for PMZL, with plans to address the adenocarcinoma through stereotactic body radiation therapy (SBRT). Unfortunately, due to deconditioning from multiple hospitalizations and a pulmonary embolism, the patient chose comfort measures and subsequently passed away.

Diagnosing PMZL can be challenging due to the absence of identifiable nodules. Reported cases have similarly required extensive investigations to reach a final diagnosis. While a direct correlation between chronic inflammation, frequent infectious pathogens, and the development of PMZL has yet to be established, a known association exists between EMZL and pathogens such as *Helicobacter pylori* in gastric involvement and *Chlamydia psittaci* in ocular adnexa.

This report highlights the difficulties in obtaining a diagnosis for PMZL and examines the various mechanisms that may have contributed to this unusual finding.

## Introduction

Marginal zone lymphoma (MZL) is a subtype of non-Hodgkin's lymphoma characterized by the proliferation of mature B cells in the outermost zone of lymphoid follicles. MZL can be further classified into three forms based on the anatomical location of the follicles: splenic, nodal, and extranodal. Extranodal marginal zone lymphoma (EMZL), or mucosa-associated lymphoid tissue (MALT) lymphoma, typically occurs in areas prone to chronic inflammation. Certain sites in the body, such as the stomach, salivary glands, ocular adnexa, and lungs, can be affected. However, existing reports on primary pleural lymphoma emphasize its rare and unusual occurrence [1,2]. Malignant lymphoma accounts for only 2.4% of primary chest wall tumors, diffuse large B-cell lymphoma, or follicular lymphoma [3,4].

When observed, MALT lymphoma commonly arises in association with chronic pleural inflammation. However, since Kodama et al. reported the first case 25 years ago, less than 20 similar instances have been documented in the literature [5].

We report for the first time in the literature a case of pleural marginal zone lymphoma (PMZL) with primary lung adenocarcinoma.

## Case presentation

**Figure 1:**
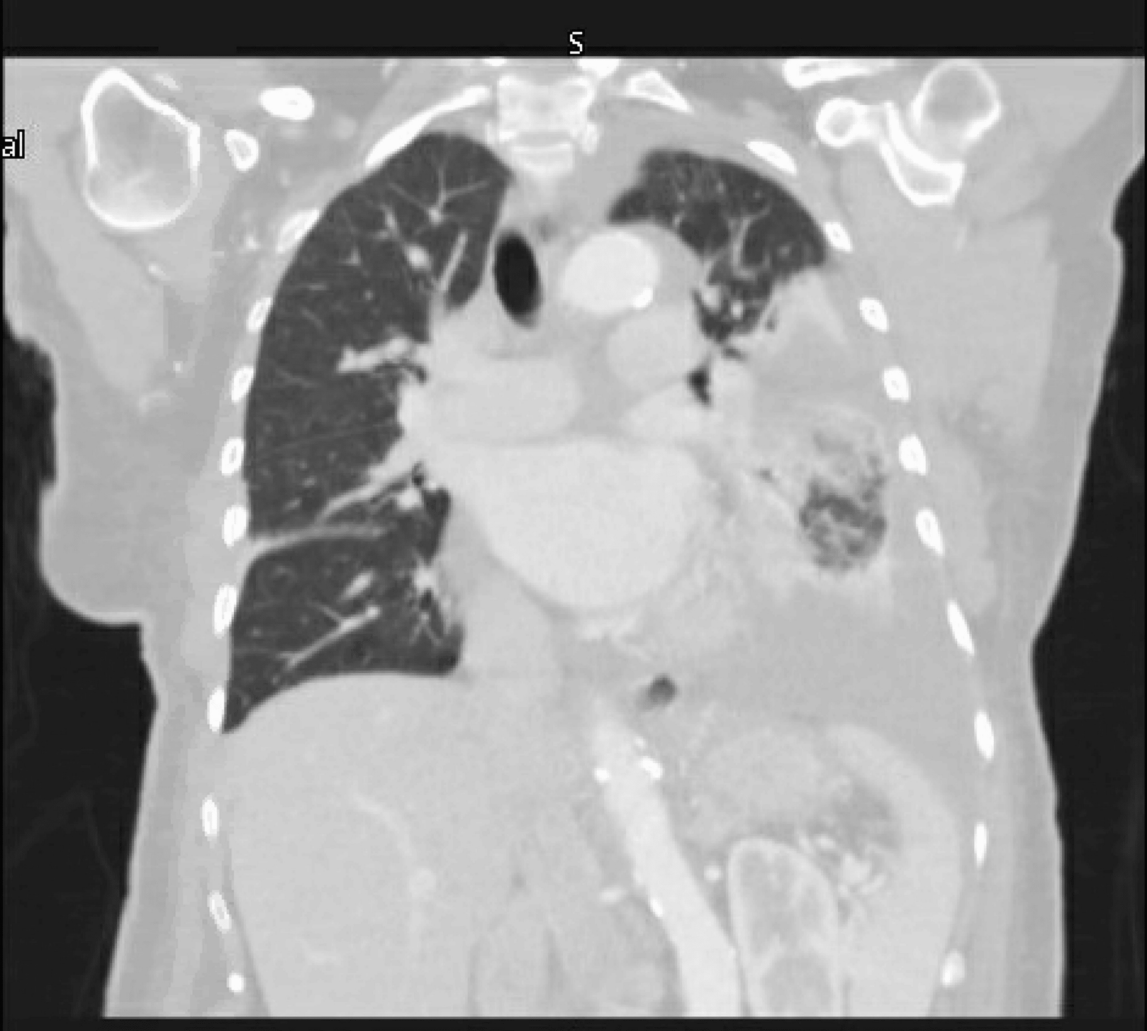
Computed tomography scan of the chest without contrast There is a mass opacification with bronchial obstruction in the left lower lobe

The patient is an 88-year-old woman, a non-smoker with a medical history that includes heart failure with reduced ejection fraction, chronic kidney disease, diabetes, and recurrent bilateral pleural effusions. Her hypertension had been well-controlled before this presentation. When she arrived at the emergency department, she reported experiencing shortness of breath, hypoxia, and a nonproductive cough. Her blood pressure was elevated, measuring 195 mmHg systolic and 100 mmHg diastolic. During the evaluation for suspected decompensated heart failure, a chest X-ray showed a left lower lobe opacity along with a moderate left-sided pleural effusion. Previous investigations, which included cytology and computed tomography (CT)-guided biopsy of the mass, had returned negative for both malignancy and infection. A subsequent chest CT scan revealed narrowing at the central lobe bronchus, accompanied by a mass in the left lower lobe and bilateral pleural effusions (Figure 1).

**Figure 2:**
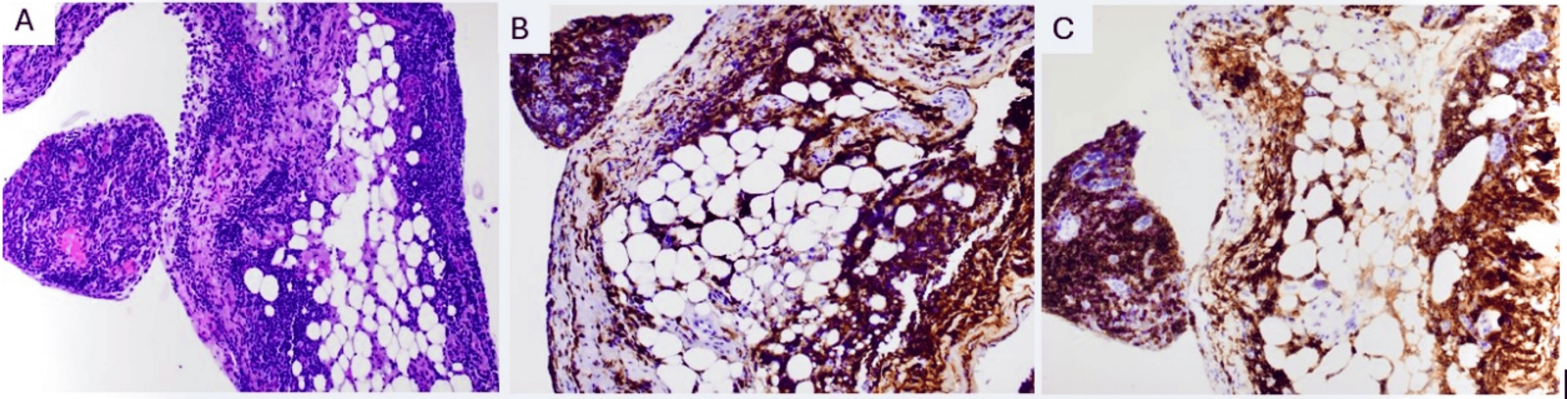
Pleural biopsy (A) H&E (200× magnification) shows bands and clusters of small lymphocytes and mesothelial cell hyperplasia. (B) The small lymphocytes are positive for CD43. (C) The cells of interest are also positive for CD20 CD, cluster of differentiation; H&E, hematoxylin and eosin

**Figure 3:**
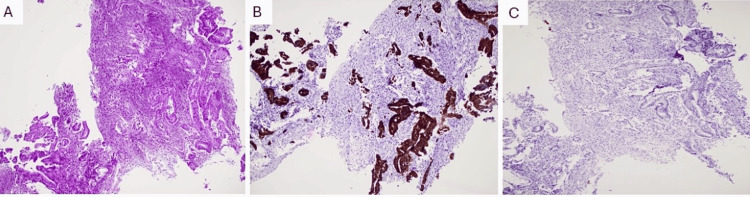
Left lung opacity biopsy (A) Hematoxylin and eosin (H&E) (100× magnification) of the lung mass biopsy shows invasive poorly differentiated adenocarcinoma. (B) The malignant cells are positive for CK7. (C) Tumor cells are negative for CK20 CK: cytokeratin

Diagnostic and therapeutic thoracocentesis were performed, which revealed a cell count characterized by lymphocytic predominance. Microbiological analysis showed a significant presence of *Aspergillus*. Following this, a thoracoscopy was conducted, and a chest tube was inserted. Random biopsies were taken from the parietal pleura, but no nodules were visualized. The left pleural biopsy indicated a monoclonal population of immunoglobulin G Kappa, which was positive for cluster of differentiation (CD) markers CD20 and CD43 but negative for CD5 and CD10, with a very low proliferation rate (Ki-67: <5%). Polymerase chain reaction (PCR) analysis was carried out, revealing a B-cell clonality panel and clonal rearrangement of the immunoglobulin heavy chain (*IgH*) and immunoglobulin kappa chain (*IgK*) genes (Figure 2) A CT-guided biopsy of the left lower lobe mass indicated a poorly differentiated adenocarcinoma with the following phenotype: CK7-positive, CK20-negative, and thyroid transcription factor-1 (TTF-1)-negative (Figure 3). It was determined to likely be of lung origin, classified as cT1b N0 M0, stage IA2. The patient was subsequently diagnosed with PMZL and poorly differentiated adenocarcinoma.

The patient's MALT lymphoma was treated with rituximab, of which she received two of four planned cycles. Given that the patient was a poor surgical candidate, stereotactic body radiation therapy (SBRT) was determined to be the best intervention for her adenocarcinoma. However, the patient became deconditioned from multiple hospitalizations and was subsequently admitted for a pulmonary embolism; she ultimately opted for comfort measures and passed away.

## Discussion

To diagnose EMZL, the National Comprehensive Cancer Network (NCCN) guidelines recommend performing an immunohistochemistry (IHC) panel. This panel should assess the patient for markers including CD20, CD3, CD5, CD10, B-cell lymphoma antigen 2 (BCL2), kappa/lambda light chains, and CD21 or CD23, with or without cyclin D1. The typical immunophenotype for EMZL is characterized by CD10-, CD5-, CD20+, CD23-/+, CD43-/+, cyclin D1-, and BCL2- follicles. This aligns with the patient's results, marking her the first reported case of PMZL in the United States and one of only nine instances documented in the literature worldwide [6].

The diagnosis of PMZL is challenging, often requiring extensive investigations before it is confirmed. Symptoms frequently point toward other conditions, which can lead to initial misdirection in diagnosis. Kodama et al. describe a 79-year-old male asymptomatic patient found to have a solid tumor of the left lung during routine screening. It was only through the resection of the lung tumor that nodules on the surrounding pleural membrane became grossly visible, and histologic examination later confirmed these to represent the PMZL [5]. For instance, a case of a 52-year-old immunocompetent woman was reported who presented with substantial bilateral pleural effusion that was regularly drained over two years. She was misdiagnosed with tuberculous hydrothorax, but then, a flow cytometry was performed on a morphologically normal lymphocyte population, which showed abnormal immunophenotypic markers consistent with PMZL [7]. Another 79-year-old man with a tobacco use disorder presented for the evaluation of chronic back pain. He was found to have a nodular pleura in a CT scan of the chest. This time, an insulation-tipped diathermic knife (IT knife) was used to resect the pleura, which facilitated the identification of abnormally appearing centrocyte-like and monocytoid-like lymphocytes. The morphology could have been otherwise obscured with conventional biopsy due to the fragility of pleural specimens [8]. In our presented case, the complex presentation of decompensated heart failure with a suspicious lung lesion concerning primary lung malignancy made the consideration of an alternative pathological cause of pleural effusion less likely. Additionally, the lymphocytes observed in the cytology appeared morphologically normal. Only the immunohistochemistry on the random pleural biopsies showed a monoclonal B-cell population and eventually the diagnosis of PMZL, which was an unexpected finding. It is also important to note that the age range for similar cases is 52-79; at 88 years old, our patient represents an outlier and sets a new upper limit in reported cases.

Evaluating the clinical context in which the patient develops this condition is worthwhile. The patient's adenocarcinoma of the lung is likely a primary neoplasm unrelated to the PMZL. However, in conjunction with the patient's history of heart failure, this speaks to a state of chronic inflammation that could have contributed to the development of the PMZL. Such a progression is in line with other reports, which have described similar presentations given a history of tuberculosis, other chronic infections of the pleura, pyothorax, and autoimmune disease [9]. The unique presence of *Aspergillus* before the PMZL in the presented case raises the likelihood of a potential association. Cases of gastric EMZL have been associated with chronic and untreated *Helicobacter pylori* infection and *Chlamydophila psittaci* with ocular adnexa. In addition, eliminating these pathogens has been therapeutic for EMZL [10]. It has been established that the chronic inflammatory conditions enabling marginal zone B-cell lymphoma development are generally of T helper 1 (TH1) type, and there is evidence that the response to *Aspergillus* is primarily TH1-mediated [11,12].

## Conclusions

This report describes the clinical presentation of PMZL in a non-smoker presenting with recurrent bilateral pleural effusion. Repeated lung biopsies failed to show any malignancy, speaking to the difficulty of this diagnosis, and there was little to explain the finding once it was established. However, a closer inspection of the few existing case reports, as well as the patient's own medical history, reveals that chronic inflammation from heart failure and possibly aspergillosis may have fostered the convenient environment for this rare development.
